# Phenobarbital use in pediatric perampanel overdose with coma, respiratory compromise

**DOI:** 10.1016/j.toxrep.2024.101809

**Published:** 2024-11-15

**Authors:** Adam Brzezinski, Cesar I. Menchaca, Shashikanth Gangu

**Affiliations:** a St. Joseph’s Children’s Hospital, Tampa, FL, USA; b University of Pittsburgh School of Medicine, Department of Critical Care Medicine, St Joseph’s Children’s Hospital, Tampa, FL, USA

**Keywords:** Perampanel, Fycompa, Overdose, Coma, Ingestion, Toxicology, Phenobarbital, Pediatric

## Abstract

Perampanel (Fycompa®) is a non-competitive alpha-amino-3-hydroxy-5-methyl-4-isoxazolepropionic acid (AMPA) glutamate receptor antagonistic antiepileptic medication used to treat generalized seizure disorders. Very little is known about the management of patients following perampanel overdose, particularly in the pediatric population. We report a case of a pediatric patient, initially presenting with increased aggression and erratic behavior, who quickly developed severe respiratory failure and prolonged coma following an intentional ingestion of between 80 and 216 mg of perampanel (1.64–4.43 mg/kg of body weight). Phenobarbital was initiated to increase the metabolism of perampanel and for seizure prophylaxis. Perampanel toxicity has been associated with a range of symptoms including bradycardia, hypotension, hyponatremia, central nervous system depression, prolonged coma, hypercoagulable state, and erratic, aggressive “zombie-like” behavior. With a reported half-life of 105 hours, no known reversal agent, and limited evidence-based management, clinicians should be prepared for prompt resuscitation and prolonged management of patients with suspected perampanel intoxication.

**Educational Objectives:**

1. Identify symptoms of perampanel overdose and consider early intubation and resuscitation for prolonged coma and respiratory failure.

2. Describe alternative methods to increase metabolization of medications with prolonged half-life.

## 1 Introduction

Perampanel (Fycompa®) is an antiepileptic drug used to treat patients with generalized seizure disorders and partial onset seizures [1], [2]. Perampanel is a non-competitive alpha-amino-3-hydroxy-5-methyl-4-isoxazolepropionic acid (AMPA) glutamate receptor antagonist and acts directly and indirectly as an N-methyl-D-aspartate (NMDA) receptor antagonist [3].

It is the first drug in its class to be approved by the Food and Drug Administration (FDA). It has been approved as an adjunctive therapy in patients 12 years of age and older with epilepsy and as monotherapy for partial onset seizures in patients as young as 4 years of age [1], [2]. The pediatric dose of perampanel starts at 2 mg daily and is titrated weekly to manage symptoms up to a max dose of 12 mg daily [1], [4]. Perampanel peaks between 1 and 3 hours and has a prolonged half-life of 105 hours, to as much as 129 h [5], [6].

There are few reports of perampanel overdose, and all case reports consist exclusively of adult patients. Several neurologic and psychiatric adverse effects are reported after overdoses of perampanel, and while some are thought to be dose-dependent, they commonly include aggression and agitation, delirium, stupor, bradycardia, hypotension, and multiple reports of “zombie-like” behavior [4], [7], [8]. In multiple case reports, it was reported that patients with high-dose perampanel toxicity, developed significant central nervous system depression leading to profound respiratory distress, with one case leading to a 14-day coma [4], [9].

To date, there is very little known about the management of patients following perampanel overdose, particularly in the pediatric population. In our patient’s case, the choice was made to initiate treatment with phenobarbital to increase the metabolism of perampanel and to prophylactically treat seizures given her underlying seizure disorder. This case report discusses the management and clinical findings of a pediatric patient who ingested between 10 and 27 times the prescribed dose of perampanel (1.64–4.43 mg/kg of body weight), which is the highest dose to body weight ingestion currently reported in the literature for a pediatric patient.

## 2 Case report

A 16-year-old female with history of epilepsy presented to the emergency department after new onset aggression and erratic behavior following an intentional ingestion of perampanel. The patient had no diagnosed psychiatric disorders prior to presentation. She had been started on 8 mg of oral perampanel daily six months prior to presentation for primary epilepsy, although the family reported known medication non-adherence. Approximately twelve hours prior to arrival, the patient ingested an estimated 80–216 mg of perampanel, between 10 and 27 times her daily prescribed dose.

On arrival at the emergency department, her initial blood glucose was 151 mg/dL, temperature 98.9 °F (37.2 °C), blood pressure was 126/70 mmHg, heart rate 132 beats/min, and respiratory rate of 22 breaths/min. Her initial laboratory testing was only significant for mild leukocytosis (13.4 WBC/mm^3^), carbon dioxide of 16 mmol/L, anion gap of 21, and a mild elevation of creatinine kinase (124 units/L). Her urinalysis and urine drug screen were negative. Serum levels of ethanol, acetaminophen and salicylates were unremarkable. Her electrocardiogram (EKG) revealed sinus tachycardia and computed tomography (CT) scan of her brain was unremarkable. The patient was severely agitated, aggressive towards staff, and incoherent on questioning. Shortly after arrival, she had multiple episodes of vomiting, and began to experience increased somnolence, associated with episodes of hypotension and bradycardia. One of Florida’s Poison Control Center was contacted, though no recommendations were made due to limited data on perampanel overdose. Given the patient’s worsening mental status, she was intubated for airway protection using etomidate and succinylcholine. She was kept sedated with a continuous propofol infusion and admitted to the pediatric intensive care unit (PICU).

On Day 1 of admission, during a sedation holiday, patient was completely unresponsive, had no spontaneous or purposeful movements, did not exhibit a cough or gag reflex, but reassuringly had pupils that were 3 mm in size, equal bilaterally, and responsive to light. She was transitioned from continuous propofol infusion to continuous fentanyl infusion for sedation given the concerns for a prolonged course based on the half-life of perampanel and the risk for Propofol-Related Infusion Syndrome (PRIS) in pediatric patients. She also continued to have episodes of bradycardia and fluid-resistant hypotension requiring the initiation of a norepinephrine infusion. The patient became febrile to 102.4 °F (39.1 °C): blood, urine, and sputum cultures were obtained. While the fever could have been contributed to seizures prior to arrival or sub-clinical seizures while sedated and intubated, the patient was started on broad spectrum antibiotics for coverage against aspiration pneumonia following multiple emeses.

On the evening of Day 1, the patient began exhibiting decerebrate posturing, with lower extremities extended and rigid, feet plantar flexed and toes curling inward. Her upper extremities were extended and rigid, arms mildly pronated, and her wrists were flexed and externally rotated. Due to concerns for subclinical seizures, she was placed on continuous electroencephalogram (cEEG), which revealed multifocal and generalized epileptiform changes. Magnetic resonance imaging (MRI) of the brain with and without contrast and magnetic resonance angiogram (MRA) were obtained, both of which were unremarkable. The patient was started on prophylactic enoxaparin (Lovenox®) as per the hospital’s deep vein thrombosis (DVT) standard of care protocol and given concerns of a similar perampanel ingestion case in which the patient developed significant pulmonary embolism burden [10]. Approximately 24 h following admission to the hospital, the patient was started on phenobarbital (4 mg/kg/day divided twice daily, intravenously and later transitioned orally) for seizure prophylaxis and with the intention of it also inducing an increase in the clearance of perampanel, which is metabolized predominantly by the cytochrome P450 in hepatocytes [11], [12]. Phenobarbital levels were obtained daily until therapeutic levels were reached (greater than 15 mcg/mL).

On Day 2, approximately 48 hours after perampanel ingestion, a perampanel level was collected as a send out laboratory study. The patient continued to have decerebrate posturing and remained unresponsive to verbal and tactile stimulation during sedation holidays. On the evening of Day 2, she had an unexplained, unexpected, spontaneous episode of aggression where she thrashed about attempting to get out of her hospital bed. She was witnessed to be gnawing at her endotracheal tube and attempted to bite a bedside nurse. This event lasted approximately two minutes, resolved spontaneously, and she was subsequently started on a continuous dexmedetomidine (Precedex®) infusion for additional sedation in conjunction with the fentanyl infusion.

On Day 3, 4, and 5 of admission, the patient had intermittent, brief episodes of spontaneous eye opening and movements, although she continued to demonstrate decerebrate posturing, unresponsiveness, and no meaningful movements. On the evening of Day 5, she developed multiple brief, self-resolving episodes of myoclonic jerks of unclear significance. Patient’s kidney functioning and transaminase levels remained within normal limits during daily trending.

On the morning of Day 6, during a sedation holiday, she was able to follow commands, and nod “yes” and “no” appropriately to questions. Her bradycardia and hypotension resolved, and she was weaned off of the norepinephrine infusion. She was started on zonisamide by pediatric neurology as her new antiepileptic medication. The patient was weaned off sedative infusions and extubated uneventfully to heated high flow, approximately 168 h after her initial ingestion.

She experienced episodes of slowed cognition and reduced strength in the days following; however, she remained calm and cooperative. Her zonisamide dose was increased and she was eventually discharged to a behavioral health facility on Day 13 of admission.

## 3 Discussion

Due to perampanel being a relatively novel medication, either used as adjunctive or monotherapy for epilepsy, there is very little known regarding the management of overdose and toxicity of the medication. Our case report is not only distinctive in that the overdose occurred in a pediatric patient – which has been very rarely reported – but that our patient experienced possibly, the highest weight-based overdose of perampanel, currently reported in the literature.

While most antiepileptics that are AMPA receptor antagonists have poor blood brain barrier (BBB) penetration and have relatively shorter half-lives, perampanel has a unique ability to penetrate the BBB when orally ingested, which is likely the cause for the profound central nervous system side effects seen in large ingestions [13], [14]. This is thought to be because perampanel is not a substrate of P-glycoprotein (P-gp) or of breast cancer resistant protein (BCRP), which are both efflux transporters in the BBB. Therefore, increased levels of P-gp and BCRP transporter levels (both thought to be associated with drug resistance in refractory seizures) are unlikely to limit access to perampanel to the brain, regardless of route of administration [14].

While many case reports of perampanel overdose reveal similar initial symptoms to those of our patient, including erratic movements, increased agitation, aggressive “zombie-like” behavior, bradycardia, hypotension, and respiratory failure, it has also been found that perampanel overdose can lead to a transient hypercoagulable state in the body and widespread pulmonary embolism, as reported by Kim and colleagues in their case study of a patient ingesting 10 times the recommended dose of perampanel [4], [7], [8], [9]. Although their patient exhibited prolonged stupor and confusion following ingestion, not requiring intubation, their patient began exhibiting worsening respiratory status on Day 3 of admission secondary to disseminated, widespread pulmonary emboli. This is theorized to occur via antagonism of the AMPA receptors that modulate platelet activation and thrombosis. High blood levels of perampanel, initially following an ingestion, block usual platelet activation and lead to a hypocoaguable state. Therefore, withdrawing from daily perampanel use or inducing metabolism of the drug could lead to a transient hypercoagulable state, such as Kim and colleagues reported [15], [10]. Thrombosis risk should be particularly monitored in pediatric patients that are immobilized for prolonged periods or that experience long-term encephalopathy.

Our patient’s ingestion of an estimated 80–216 mg of perampanel resulted in a perampanel level of 1200 ng/mL at approximately 48 hours following ingestion. Based on the pharmacokinetic/pharmacodynamic data provided by LabCorp Data, daily administration of 6 mg perampanel results in peak plasma concentration averaging 460 ng/mL at 1.3 h post dose and a peak concentration following a single 12 mg dose of perampanel averaged 800 ng/mL. Unfortunately, our medical team was unaware of the possibility of sending a perampanel level and it was not an orderable test from our electronic medical system, which is why no such level was obtained at presentation. We later found that this was a requested special send out lab test that is not typically commonplace to order. We did not trend serum perampanel levels throughout patient’s admission.

Because of the significant amount of perampanel ingested, the choice was made to treat our patient with phenobarbital. Not only for seizure prophylaxis, but with the intention of theoretically inducing an increase in the clearance of perampanel. An in vitro study involving perampanel metabolism demonstrated that perampanel is predominantly metabolized by the cytochrome P450 in hepatocytes, specifically by the cytochrome P450 isotype CYP3A4/5 [11], [12]. This is why we did not attempt hemodialysis on our patient. We suspect that based on the estimated dosage that our patient ingested, phenobarbital therapy may have been helpful in increasing the hepatic metabolization of perampanel.

Following erratic and aggressive episodes in the emergency department and in the PICU, our patient was treated with continuous dexmedetomidine infusion. Case reports, such as the one Morsi and Katz describe, have reported great success with the use of dexmedetomidine infusions in treating the severe agitation and “zombie-like” behavior in patients with perampanel toxicity[16], [7].

Many case reports reveal that while acute perampanel overdose may not initially cause end organ damage or metabolic disturbance, it can cause prolonged central nervous system depression with secondary respiratory failure [9]. Due to the long 105-hour half-life, early recognition and supportive care are paramount in the survival of patients. Table 1

**Table 1.** Treatment recommendation guidelines for pediatric perampanel overdose.

Neurological Considerations	Obtain Neurology consultation Obtain continuous EEG monitoring Obtain MRI/MRA/MRV to rule out intracranial process unrelated to ingestion Sedation with dexmedetomidine infusion Initiate phenobarbital therapy to induce hepatic clearance
Cardiovascular Considerations	Norepinephrine infusion for hypotension EKG and echocardiogram
Respiratory Considerations	Early airway protection with intubation
Gastroenterological Considerations	Nasogastric/orogastric tube feedings while sedated, intubated
Hematological/Oncological Considerations	Initiate anti-coagulant therapy for prolonged immobilization, or comatose status
Infectious Disease Considerations	Initiate antibiotic therapy if there are concerns for superimposed infection

This treatment guideline is a recommendation following the treatment of our patient in the pediatric intensive care unit. The treatment guidelines described are limited by the scarce resources available and literature published on perampanel overdose. While early recognition and supportive care are the mainstay of therapy, future treatment guidelines would benefit from further research on perampanel and the effects of acute ingestion and overdose.

## 4 Conclusion

This case report is one of the first of its kind regarding severe perampanel toxicity in a pediatric patient. There is little known about the management of perampanel toxicity. With reports of mild agitation and aggression, ranging to severe central nervous system depression and prolonged comas, it is necessary to identify perampanel overdose quickly and intervene rapidly if patients deteriorate. Perampanel should be considered in the differential diagnosis when polypharmacy ingestion is seen in the emergency department, particularly with patients with a history of seizure disorders. Additional studies are needed to determine toxic doses in different age populations, as well as determining possible methods to counteract perampanel’s extended half-life, such as the use of phenobarbital in the intensive care setting. By understanding more about perampanel, health care providers can ensure optimal outcomes following perampanel overdose in both adult and pediatric populations.

## Financial Disclosure

The authors have indicated they have no financial relationships relevant to this article to disclose.

## Funding

This work was supported by St. Joseph’s Children’s Hospital.

## CRediT authorship contribution statement

**Adam Thomas Brzezinski:** Writing – review & editing, Writing – original draft, Conceptualization. **Cesar Menchaca:** Writing – review & editing, Conceptualization. **Shashikanth Gangu:** Writing – review & editing, Conceptualization.

## Declaration of Competing Interest

None declared.

## Data Availability

No data was used for the research described in the article.
